# To scan or not to scan? Examining the controversial issue of performing neuroimaging in adolescent patients presenting to a tertiary psychiatric inpatient unit

**DOI:** 10.4102/sajpsychiatry.v26i0.1383

**Published:** 2020-02-06

**Authors:** Zureida Khan, Anusha Lachman

**Affiliations:** 1 Department of Psychiatry, Faculty of Medicine and Health Sciences, Stellenbosch University, Cape Town, South Africa

**Keywords:** imaging, psychiatry, clinical utility, neuroimaging, adolescents, psychiatric presentation

## Abstract

**Background:**

Imaging techniques such as computerised tomography (CT), magnetic resonance imaging (MRI) and single photon emission computed tomography (SPECT) scans are used in various clinical and diagnostic neuropsychiatric assessments. However, these investigations may be costlier when compared to their clinical utility.

**Aim:**

To examine the clinical utility of neuroimaging in an acute adolescent psychiatric inpatient population admitted to Tygerberg Hospital between January 2012 and December 2013.

**Setting:**

The study was conducted at a tertiary level adolescent psychiatric inpatient unit at Tygerberg Tertiary Hospital, Parow, Cape Town, Western Cape, South Africa.

**Method:**

A retrospective chart review was conducted to gather data from 125 inpatient adolescents who had neuroimaging performed during admission. Clinical information was obtained from folders and collated with neuroimaging data. The Pearson Chi-squared test was used to test for correlations between clinical variables and the outcomes (abnormalities) of CT scans. There were too few MRI or SPECT scans to warrant statistical testing for these modalities.

**Results:**

Out of the total CT scans performed (*n* = 120), 11 (9.2%) were clinically significant or pathological. Five cases (4.2% of all CT scans) resulted in a change in diagnosis and management. There was no association between clinical variables and clinically relevant CT abnormalities (*n* = 11). There were three MRI abnormalities (30%), with two resulting in changes in management. Single photon emission computed tomography scans revealed abnormalities in all 10 cases.

**Conclusion:**

Routine neuroimaging in this population of psychiatric adolescents has high clinical utility. However, the decision to conduct structural neuroimaging should be guided by good clinical assessment. Single photon emission computed tomography scanning is useful for detecting underlying neurophysiological abnormalities in patients presenting with psychiatric and behavioural symptoms to potentially aid diagnosis and for interventional purposes.

## Introduction

Neuroimaging is used to detect brain tumours, structural lesions causing psychosis and to differentiate depression from neurodegenerative disorders in psychiatry.^1^ Structural brain imaging is often performed in adolescents presenting with first episode psychosis (FEP) and other acute psychiatric symptoms, usually to exclude any underlying treatable and potentially reversible neurological pathology. However, this practice remains controversial as no clear local guidelines exist to inform routine requests for imaging in these adolescents. Numerous international studies suggest that neuroimaging should not be part of the routine assessment in psychiatric presentations.^2,3^ Furthermore, the National Institute for Health and Care Excellence (NICE) Guidelines of 2008 recommend that neuroimaging should not be requested routinely during initial investigations for the management of FEP patients.^4^ Several other studies suggest that certain clinical variables are associated with neuroimaging abnormalities and would indicate imaging as part of routine assessment.^5,6^

Studies concerning the clinical applications of brain imaging in psychiatry have, for the most part, been limited to the use of structural imaging, such as computerised tomography (CT) and magnetic resonance imaging (MRI), to rule out neurologic disorders in the differential diagnosis of major mental illnesses.^7^ The role of functional neuroimaging, such as single photon emission computed tomography (SPECT), in psychiatry has been used predominantly to investigate pathophysiology and interventional and treatment targets in adult populations. Recently, SPECT has received attention as a potentially clinically useful modality in neuropsychiatry.^8,9^

At the adolescent psychiatric inpatient unit at Tygerberg Hospital (TBH; Cape Town, South Africa), imaging techniques, such as CT, MRI and SPECT scans, are used in a variety of clinical and diagnostic neuropsychiatric assessments. However, because South Africa is considered a low- to middle-income country and has significant resource limitations within the health care system, there are concerns about financial costs in relation to the clinical utility of neuroimaging in the local context. Indeed, clinical utility is a significant factor in clinical and corporate governance.^10,11^ According to Williams et al., the issues to consider with respect to clinical utility are clinical yield, radiation exposure and cost of neuroimaging.^12^ The clinical utility of these neuroimaging investigations in the South African adolescent population group is unknown. Such information would be useful to assist in the development and management of safe and cost-effective neuroimaging investigations, both in our child and adolescent inpatient units and in other facilities. To our knowledge, there are no similar studies available in the literature, which examine the clinical utility of neuroimaging in adolescent populations in low- to middle-income countries. Therefore, the aim of this study was to determine the clinical utility of the different neuroimaging studies requested by our unit. To do this, we assessed the clinical yield of abnormalities detected by different modalities, the impact of these abnormalities on management and the possible associations between these abnormalities and various clinical variables.

## Research methods and design

### Study design

We performed a retrospective chart review of patients referred to the adolescent psychiatric inpatient unit at TBH (Cape Town, South Africa) between 01 January 2012 and 31 December 2013.

### Study setting

Tygerberg Hospital is a tertiary academic hospital situated in the northern suburbs of the city of Cape Town and serves a low- to middle-income population of 2.6 million people from a large drainage area including the Northern Metro sub-districts, Khayelitsha – north of Spine Road, Eastern Tygerberg, West Coast, Cape Winelands and the Overberg rural districts. The adolescent psychiatric inpatient unit provides services for children and adolescents up to the age of 18 years. Patients are admitted with a variety of psychiatric presentations requiring diagnostic assessment and intervention. The unit consists of 16 beds, 11 of which are reserved for acute admissions. A further five beds are reserved for therapeutic or diagnostic admission.

### Study population

A total of 201 adolescents (12–18 years) were admitted between 01 January 2012 and 31 December 2013. Folders with missing information (such as no record of physical examination and incomplete medical histories) were removed. Patients who had no neuroimaging performed were also excluded. The final sample consisted of 125 patients.

### Data collection

Clinical and demographic information was extracted from the patient charts and collated on a Microsoft Excel spreadsheet. Patients who underwent neuroimaging were then identified, and neuroimaging data were accessed using the radiology Picture Archiving and Communication System (PACS) at TBH. Data for patients who had neuroimaging studies performed at other institutions were obtained electronically by contacting these institutions and requesting the neuroimaging results and reports. The initial neuroimaging reporting was completed by various radiologists at TBH, and two scans were reported by a radiologist at the Department of Radiology at Groote Schuur Hospital (Cape Town, South Africa). The number of scans totalled 140, as some patients were scanned using multiple modalities. All neuroimaging results were reviewed in consultation with a single senior radiologist at TBH to verify the presence of abnormalities and the clinical relevance of each abnormality. The clinical impact of these abnormalities on management was assessed by re-examining the clinical charts of the respective patients to assess what the treating clinician, at the time of admission, had decided in terms of management and diagnosis.^13^ On examination of the clinical charts, it was noted that psychiatric disorders were diagnosed according to the Diagnostic and Statistical Manual of Mental Disorders-4 (DSM-4).^13^ Diagnoses made prior to 2013 were reviewed and reconsidered using DSM-5 criteria.

### Data analysis

Continuous variables were summarised as mean and standard deviation (SD) or median and 25th–75th percentiles, while nominal variables were summarised as counts and percentages. The Pearson Chi-squared test was used to test for correlations between clinical variables and the outcomes (abnormalities) of CT scans. There were too few MRI or SPECT to warrant statistical testing for these modalities. All analyses were performed using STATISTICA version 10 (StatSoft Inc, 2011), and the level of significance was set at *p* < 0.05.

### Ethical considerations

Ethical approval was obtained from the Health Research Ethics Committee of Stellenbosch University (ref #S13/10/191), and a waiver of informed consent was granted. The study was also approved by the management of Tygerberg Hospital. All data were anonymised to ensure privacy and confidentiality of participants’ personal information, and each patient was assigned a unique identifier.

## Results

Our sample of 125 adolescents included 74 (59.2%) men and 51 (41.8%) women. The mean age of patients was 15.98 years (SD: 1.17). The majority presented with psychosis and 71 (59.71%) had a history of substance use disorder (Table 1).

**TABLE 1:** Clinical characteristics of patients (*n* = 125).

Clinical characteristics	*n*	%
Psychosis	80	66.13
HIV positive	8	66.70
Substance use disorder	71	59.71
Head injury	10	8.33
Epilepsy	7	5.83
Syphilis	2	1.67
Catatonia	11	9.16

Most patients (96%) underwent CT imaging (Table 2). Only 8% received MRI scans and a further 8% received SPECT scanning. Of all CT scans performed (*n* = 120), 19 (15.8%) detected abnormalities and only 11 (9.2%) were clinically significant or pathological. All patients who had abnormal scans had normal clinical neurological examinations according to the clinical records.

**TABLE 2:** Neuroimaging modalities and detected abnormalities.

Imaging modality	Number of patients scanned (*n* = 125)†		Abnormal scans‡					
	*n*	%	Total		Clinically significant		Clinically non-significant	
			*n*	%	*n*	%	*n*	%
CT	120	96.0	19	15.8	11	9.2	8	6.7
MRI	10	8.0	3	30.0	3	30.0	0	0.0
SPECT	10	8.0	10	10.0	10	10.0	0	0.0

CT, computerised tomography; MRI, magnetic resonance imaging; SPECT, single photon emission computed tomography.

† , Some patients were scanned using multiple modalities.

‡ , Percentages are calculated relative to the total number of scans for each modality.

Of the 11 clinically significant abnormalities detected by CT scans, five cases showed generalised, age-inappropriate atrophy. None of these abnormalities led to a change in diagnosis and/or management. Only four cases (3.3% of all CT scans) resulted in a change in diagnosis and management, and one case resulted in a change of only management (Table 3).

**TABLE 3:** Clinical features, diagnosis and management of six patients with clinically significant computerised tomography abnormalities.

Provisional diagnosis	Relevant medical history	CT scan finding	Change in diagnosis and/or management
Psychosis secondary to substance use	Substance use disorder	Temporal haemorrhage	Referred to neurology
Suicidality and depression	Substance use disorder	Brain swelling	Referred to neurology
Cognitive disorder	Patient has tuberous sclerosis	Mass lesion	None
Somatoform disorder versus organic pathology	History of head injury; HIV positive, syphilis reactive	Acute hydrocephalus secondary to neurocysticercosis	Referred to neurology
Psychosis secondary to substances	Substance use disorder	Low-grade white matter changes	Diagnosed as psychosis secondary to general medical condition
Psychosis secondary to medical condition	HIV positive	Old bilateral frontal and occipital infarcts	Referred to internal medicine
Psychosis secondary to medical condition	Head injury, HIV positive	Generalised, age inappropriate atrophy	None
Psychosis secondary to substances	Substance use disorder	Generalised, age-inappropriate atrophy	None
Psychosis	None	Generalised, age-inappropriate atrophy	None
Psychosis	None	Generalised, age-inappropriate atrophy	None
Psychosis	Substance use disorder	Generalised, age-inappropriate atrophy	None

CT, computerised tomography.

Results of the Pearson’s Chi-square test showed no significant associations between any of the clinical variables (HIV, head injury, birth trauma, syphilis, substance use disorder, epilepsy and catatonia) and the presence of a CT abnormality (*n* = 19). Furthermore, there was no association between these clinical variables and clinically significant or relevant CT abnormalities (*n* = 11).

Of the 10 MRI scans conducted, only three (30%) showed abnormalities. Diagnosis and management were changed in one case, and management alone was changed in another case (Table 4)

**TABLE 4:** Clinical features, diagnosis and management of three patients with clinically significant magnetic resonance imaging abnormalities.

Provisional diagnosis	Relevant medical history	MRI finding	Change in diagnosis and/or management
Cognitive disorder	Tuberous sclerosis	Multiple subcortical tubercles, right ventricular lesion giant cell astrocytoma	Motivation for the treatment of giant cell astrocytoma
Psychosis secondary to substances	Substance use disorder	T2 hyperintensities in the centrum semiovale and periventricular areas bilaterally	Diagnosis changed from substance-induced psychosis to psychosis secondary to GMC
Cognitive disorder	Tuberous sclerosis	Non-progressive tuberous lesions	None

MRI, magnetic resonance imaging; GMC, general medical condition.

All the SPECT scans conducted revealed abnormalities; however, diagnosis and management changed for only one case (Table 5). All other abnormalities showed non-specific perfusion abnormalities.

**TABLE 5:** Clinical features, diagnosis and management of patients with clinically significant single photon emission computed tomography abnormalities.

Provisional diagnosis	Relevant medical history	SPECT finding	Change in diagnosis and/or management
Cognitive disorder	Tuberous sclerosis	Decreased perfusion in frontal lobes	None
Psychosis secondary to substances	Substance use disorder	Decreased front temporal perfusion	None
Psychosis	None	Decreased cortical perfusion	None
Delirium	Neurocysticercosis	Decreased frontotemporal perfusion	None
Cognitive disorder	Tuberous sclerosis	Decreased cortical and subcortical perfusion	None
Delirium	None	Decreased perfusion in the frontal lobes	None
Psychosis secondary to medical condition	Systemic lupus erythematosus	Decreased anterior frontal cortex	Neurolupus
Psychosis secondary to substances	Substance use disorder	Decreased perfusion to cortex and cingulate area	None
Psychosis secondary to medical condition	HIV positive	Decreased perfusion to frontal, parietal and temporal lobes	None
Somatoform disorder versus organic pathology	HIV positive; head injury; rapid plasma regain reactive	Patchy decreased uptake in frontal cortex	None

SPECT, single photon emission computed tomography.

## Discussion

We evaluated the clinical utility of different neuroimaging modalities using an acute adolescent psychiatric inpatient population at TBH. Findings from our study indicate that CT imaging is the preferred modality for diagnostic neuroimaging in our setting. The overall yield of CT abnormalities detected was moderate (*n* = 19, 15.8%), and 11 scans showed abnormalities that were clinically significant. Clinical significance of detected abnormalities was determined by discussing each of the findings with experts from the Department of Radiology at TBH. In addition, of these 11 abnormalities, only five (4.2%) led to changes in management. Although Berk concluded that several clinical variables can be correlated with CT abnormalities, such as neurological abnormality, diagnosis of delirium or dementia, electroencephalogram (EEG) abnormality, older age, prior head injury, substance use and neuropsychological test abnormality,^5^ we did not find any significant associations between clinical variables and detection of CT abnormalities.

Numerous studies suggest that neuroimaging should not be part of the routine assessment in psychiatric presentations owing to low clinical yield.^2,3,14^ Overall, it is evident from our investigation that the routine use of CT scans for psychiatrically ill adolescents is potentially contentious when considering clinical yield alone (15.8%). However, for those cases where management was changed, this led to significant changes in outcome. These cases in conjunction with the clinical yield therefore result in a high clinical utility.

Both CT scanning and MRI are structural scanning modalities. Magnetic resonance imaging has the advantage of minimal radiation exposure as opposed to CT.^15,16^ Magnetic resonance imaging also has higher sensitivity for detection of ischemic vascular disease and anatomical abnormalities in intracerebral soft tissue,^17^ particularly smaller lesions at the base of the skull (such as orbitofrontal and medial temporal areas).^18^ However, Khandanpour et al. concluded that there was no significant difference between MRI and CT imaging in detecting organic diseases potentially responsible for FEP.^19^ Computerised tomography imaging also has the advantage of being available in most hospitals worldwide and has a relatively low operating cost.^18^ The indications for MRI in our patients were related to the increased sensitivity of MRI to detect intracerebral soft tissue abnormalities and better define anatomical lesions. In our study, 10 MRI scans were performed. Only three MRI scans detected abnormalities, two of which led to changes in management or diagnosis. The change in management entailed motivation for treatment of giant cell astrocytoma associated with tuberous sclerosis for symptom relief and control of epilepsy. The change of diagnosis and management entailed the identification of an underlying vascular pathology in a patient presenting with psychosis and a history of substance use disorder. Magnetic resonance imaging is costlier than CT imaging, and its use should be limited for specific clinical purposes.

All SPECT scans revealed abnormalities, but these typically reflected physiological abnormalities and did not necessarily correspond with structural abnormality. The original indication question for the SPECT scan was answered in only four cases. Very specific questions were asked when requesting SPECT scans. These included situations in which diagnostic dilemmas were addressed, such as the role of perfusion patterns to confirm a diagnosis of cognitive impairment versus pre-existing intellectual disability. Other requests involved assessment of pre- and post-medication response in specific neuropsychiatric disorders. While there were subtle perfusion changes and deficits reported, these were not to be viewed in isolation, but rather required further clinical and neuropsychological testing. Single photon emission computed tomography scanning is a useful modality to examine underlying neurophysiological abnormalities in patients with psychiatric presentations to potentially aid diagnosis and intervention.

This study has several limitations. The study was limited to one site and the sample size was small, which limits the generalisability of our findings. Furthermore, our findings might be limited by the quality and consistency of the information available from inpatient folders. In addition, there is variability in terms of radiological centres conducting the different neuroimaging studies, as well as different radiologists reporting on the studies. This could lead to inter-observer variability. Selection bias is an additional limitation in this study, in that only patients with the most severe symptoms are admitted to a tertiary inpatient psychiatric unit. To better examine specific clinical variables associated with CT abnormalities, prospective studies examining neuroimaging in specific clinical populations are recommended.

## Conclusion

Overall, we found that the clinical utility of routine neuroimaging in this population is high when considering clinical yield in conjunction with cases where the abnormalities resulted in a change of management and subsequently a change of outcome. However, the decision to conduct structural neuroimaging should be guided by good clinical histories and examination, and there should also be an increased index of suspicion in patients with positive medical histories and histories of substance use disorder (Box 1). Further studies should focus on specific clinical populations to examine associations of structural neuroimaging abnormalities with specific medical conditions. When clinically indicated, SPECT scanning is a useful modality for examining underlying neurophysiological abnormalities to potentially guide diagnosis, to suggest targets for treatment and to monitor intervention.

BOX 1 Recommendations for the clinician.

1. Clinical utility of routine imaging in adolescents presenting with acute psychiatric symptoms is high in this tertiary setting when the definition of clinical utility includes clinical yield and impact on management.
2. Consider the following screening prior to the imaging request:
  - Full neurological and physical examination
  - History of medical or physical illness or injury
  - History of neuropsychiatric disorders
3. Single photon emission computed tomography is not of routine diagnostic utility for the evaluation of paediatric neuropsychiatric disorders, but patterns of typical versus atypical development or perfusion may elucidate pathologic mechanisms and suggest targets for intervention.
4. In a resource-constrained country, the rational request for expensive investigations should be strongly considered within the balance of clinical evidence.
